# Using a brief family-based DBT adjunct with standard FBT in the treatment of Anorexia Nervosa

**DOI:** 10.1186/2050-2974-3-S1-O39

**Published:** 2015-11-23

**Authors:** Annaleise Robertson, Colleen Alford, Andrew Wallis, Jane Miskovic-Wheatley

**Affiliations:** 1 The Children's Hospital at Westmead, Australia

## 

The outpatient program at The Children's Hospital at Westmead (CHW) Eating Disorder Service uses Maudsley family-based therapy (FBT) as the main therapeutic model. For families that experience a high level of distress, avoidance of conflict or emotional expression, or if the young person is engaging in high risk behaviour like self-harm or suicide attempts, the parents ability and confidence to persist with refeeding is often severely undermined. In practice this reduces the efficacy of standard FBT, leading to poor engagement, multiple inpatient admissions, longer treatment and drop-out. The team at CHW are trialling a brief 6 session adjunct based on the core principles of Dialectical Behaviour Therapy (DBT), teaching the young person, their parents and siblings basic skills to engage in mindfulness, distress tolerance, emotion regulation and effective interpersonal relationships. These DBT skills have been adapted to a framework that maintains the core principles and structure of FBT. This oral presentation will discuss the family-based DBT adjunct and its implementation, including session outlines and a case example. Initial data on its effectiveness for patient and parent experience may also be discussed.

